# Sickness absence > 14 days following sport-related traumatic brain injuries: a nationwide register-based study in Sweden

**DOI:** 10.1186/s12889-025-23711-2

**Published:** 2025-07-23

**Authors:** Christian Oldenburg, Linnea Kjeldgård, Helena Stigson, Emilie Friberg

**Affiliations:** 1 Division of Insurance Medicine, Department of Clinical Neuroscience, Karolinska Institutet, Stockholm, SE–171 77 Sweden; 2 The Swedish National Road and Transport Research Institute (VTI), Linköping, Sweden; 3 Folksam Insurance Group, Stockholm, Sweden

**Keywords:** Traumatic brain injury, Concussion, Return to work, Sick leave, Population-based, Insurance medicine, Sports medicine, Real-World data

## Abstract

**Background:**

Sport-related traumatic brain injuries (SR-TBI) have received increasing concerns regarding potential long-term consequences. For adults in the general population, one of these consequences is inability to resume daily activities, most notably sickness absence (SA) from work. The aim of this study was to investigate how often SR-TBI is followed by a registered period of SA.

**Methods:**

A nationwide register-based study was conducted. Source population: working aged individuals (18–63 years), living in Sweden during 2014–2016. Using the national patient register we included patients with a TBI diagnosis with the ICD-10 activity code ‘while engaged in a sporting activity’, excluding those with a TBI diagnosis in the preceding 365 days. Information on type of injury (e.g., concussion), cause of injury (e.g., falls, strikes by objects) and received healthcare (outpatient and/or inpatient) was collected. Basic sociodemographic variables were fetched from Statistics Sweden (age, sex, education level, marital status, type of living area, country of origin, receiving income from work). The Swedish Social Insurance Agency provided data on SA episodes lasting longer than 14 days. We calculated the incidence of new SA and estimated risk ratios (RRs) with 95% confidence intervals using modified Poisson regression (log link, robust variance) for both crude and multivariable models. Multinomial logistic regression assessed factors associated with SA duration (< 30 days, 30–90 days, > 90 days).

**Results:**

2826 individuals were identified. The majority (91%) had suffered a concussion. A new SA was found for 7% of the individuals with concussion and 32% for those with other intracranial injuries. Higher risk of a new SA was observed for other intracranial injuries compared to concussion (RR 2.76, 95% CI 2.15–3.53), inpatient care versus outpatient only (RR 1.92, 95% CI 1.50–2.46), and transport accidents versus strikes by living forces (RR 2.14, 95% CI 1.43–3.19). Higher odds for longer spells (> 90 days) were found for those with other forms of intracranial injuries than concussion, OR 10.20 (5.80-17.95) and those who had received inpatient healthcare, OR 3.68 (2.10–6.46).

**Conclusion:**

The vast majority of all SR-TBIs are concussions and seldom followed by a new SA. For patients with other types of SR-TBI and for those who required inpatient healthcare a new, and often longer SA were more frequent. Additionally, injuries from transport-accidents (e.g., horseback riding, bicycling) elevated the risk of a new SA, potentially indicating the presence of other physical injuries.

## Background

Although participation in sports is generally considered good for overall health [1, 2], there is also a risk for physical injury, including traumatic brain injury (TBI). A sport-related TBI (SR-TBI), which includes concussions as a particularly mild form of TBI, can be defined as a brain injury induced by biomechanical forces during a sporting activity, practiced at recreational, elite, or professional level [3]. The injury can occur in all sports, but the incidence is highest in contact sports like ice hockey and football or in sports using modes of transportation, such as equestrian sports or bicycling [4]. Research in concussion biomechanics has suggested a threshold at approximately 60–100 g [5], and that both linear and rotational forces significantly influence the occurrence of concussion, with rotational acceleration particularly linked to brain tissue deformation and injury severity. Thus, increase in and the nature of forces applied lead to more significant impacts on the brain [6]. Studies have shown a large variation when the impact to the athlete’s head was measured (e.g., by using special helmets) and correlated with clinical signs of concussion. More specifically, for each recorded concussion at a certain impact magnitude, many hits of equal magnitude did not result in concussion. This observation suggests the existence of individual differences in injury thresholds [7].

TBI is a very common injury, with an estimated worldwide incidence of 70 million new cases annually [8], and as many as 20% of these are believed to have originated in a sporting activity [9]. In general, more men than women suffer a SR-TBI [10, 11], however there is evidence that women have a higher risk of sustaining a SR-TBI when practicing in comparable sports [12]. Especially concussions, which are classified as a mild TBI [13], have raised growing concerns among sport communities. These concerns addresses different clinical questions, such as long-term health risk for repeated concussions [14], guidelines for rest and return-to-play after injury, and how to handle athletes with persistent symptoms beyond the expected time frame for normal clinical recovery [3].

Athletes often experience relatively rapid clinical recovery. Athletes who have sustained a SR-TBI have a swift resolution of associated symptoms [15] and cognitive deficits [16, 17] within two weeks following injury [18]. However, these are findings on group-level, which can obscure individual or subgroup differences [19]. Indeed, there is a growing body of evidence that a significant number of younger athletes (high school and college students) have a much slower clinical recovery [20]. This is consistent with the vast research literature on adults in the general population who have sustained a mild TBI, not specifically in a sporting activity, where 20–40% report persistent post-concussion symptoms three to six months after injury [21]. Available literature also frequently reports that females tend to have a longer trajectory to recovery and worse outcome than males, regarding lingering symptoms [22, 23].

The frequency with which adult individuals experiencing slower symptom resolution struggle to resume daily activities, particularly work, is attracting increasing research interest, although with methodological challenges. Theses methodological challenges were highlighted in a systematic review on return to work and employment after mild TBI. The review could only include four studies out of a pool of 101 studies with prognostic data that the overall review group (The International Collaboration of Mild Traumatic Brain Injury Prognosis) had selected [24]. The authors of the review highlighted several challenges, including unclear definitions of return to work, small sample sizes, only single-site studies, and varying compensation systems. Based on these four studies however, they concluded that most injured individuals return to work within three to six months, but that 5 to 20% experience persisting problem one to two years after injury [25].

In 2018, Bloom et al. included 14 studies in their systematic review on return to work after mild TBI [26]. They concluded that more than half of mild TBI patients return to work by 1 month, and around 80% 6 months after injury. However, they acknowledged that the studies suffered from poor internal validity and several possible biases, such as selection bias, use of self-report data, and lack of control of pre-injury status. These two reviews focused exclusively on mild TBI, which accounts for approximately 70–90% of all TBIs [27]. A systematic review of studies involving patients with moderate and severe TBI found that only 41% had returned to work two years post-injury [28].

The challenges mentioned above can be remediated by use of official register data. Using nationwide registers, a group of injured individuals of adequate size can be followed with virtually no dropouts. Individuals will be selected based on medical records, established by physicians. Sickness absence (SA) can be traced through registered paid compensation rather than relying on self-reported data. Hypothetically individuals who suffer a SR-TBI may be drawn from an active, healthier and more health-conscious part of the population suggesting the possibility for a unique recovery and faster return-to-work pattern compared to the wider TBI-population. Our aim for this current study is thus to examine SA following SR-TBI among adults in the general population using nationwide official registers.

## Method

### Study design and population

We conducted a prospective cohort, population-based register study to assess SA in working-aged individuals who experienced a TBI during a sporting activity. The study considered all individuals, aged 18 to 63 years, who lived in Sweden from 2014 to 2016, as eligible participants. We used microdata from nationwide administrative registers to identify individuals who received inpatient or specialised outpatient healthcare (including emergency department visits) due to a TBI sustained during a sporting activity.

### Data sources

In this study, we obtained micro data from five nationwide registers, linked at the individual level using the unique personal identity number assigned to all residents in Sweden [29]. We used the National Patient Registers for information about inpatient healthcare and specialised out-patient healthcare kept by the National Board of Health and Welfare, to identify the study population and extract additional medical information related to the injury. Additionally, we used the Cause of Death Register from the same authority to identify individuals who had died within the first 30 days after their injury. It is important to note that the national patient register holds information on all in- and specialised outpatient healthcare, that is, regarding secondary healthcare, but not information regarding primary healthcare (i.e., visits to general practitioners or family doctors etc.).

Data from the Longitudinal Integration Database for Health Insurance and Labour Market Studies (LISA), kept by Statistics Sweden [30], were used to identify the source population, which included all individuals aged 18 to 63 residing in Sweden during the three years 2014 to 2016. Statistics Sweden performed the linkage and hold the key. From LISA, we also extracted sociodemographic information, such as age, sex, educational level, country of birth, type of living area in terms of degree of urbanization, marital status, and presence of income from work.

Finally, we utilized the Micro Data for Analysis of the Social Insurance (MiDAS) from the Swedish Social Insurance Agency for information on the dates and duration of sickness absences and disability pensions.

### Inclusion criteria

We used the diagnostic information from the patient registers, classified according to the International Statistical Classification of Diseases and Related Health Problems, tenth revision, ICD-10 [31]. We identified all cases with a main diagnosis of Intracranial injury (S06) and subdivided these into two types of injury: concussion (S06.0) and other intracranial injuries. The latter category includes, but is not limited to, cerebral oedema, haemorrhage and focal brain injury (codes S06.1 to S06.9). To narrow down to only sport-related injuries we used the accompanying codes for external causes for morbidity and mortality used in ICD-10. Specifically, we used the activity code ‘while engaged in sports activity’ as a filtering criterion.

We then classified the cases into four groups according to the registered cause of injury: transport-accidents (codes V01-V99), e.g., a head injury sustained from a fall during a horseback riding session; falls (codes W00-W19), e.g., a concussion from a fall on ice in ice-hockey; exposure to inanimate mechanical forces (codes W20-W49), e.g., a hockey player hit by a puck, which we subsequently refer to as “strikes by objects”; and exposure to animate mechanical forces (codes W50-W64), e.g., a football player’s head injury from an opponent’s tackle, which we refer subsequently to as “strikes by living forces”.

### Exclusion criteria

To ensure that the TBI was, in fact, newly acquired, we excluded individuals with prior healthcare visits for a TBI (S06) in the 365 days prior to the injury date. Those who had healthcare visits for TBI that occurred more than one year before were categorized as having a prior TBI.

### Outcome

The primary outcome of this study was the receipt of sickness absence benefits from the Swedish Social Insurance Agency. According to Swedish laws and regulations, residents aged 16 years and older, who have income from work, unemployment, or parental leave, are eligible to apply for SA benefits if they have a disease or an injury that leads to a reduction in work capacity.

The first day of a SA-spell is a qualifying day and not reimbursed, except for self-employed individuals who can choose to have more qualifying days in exchange for a tax reduction. If the SA-spell lasts for more than seven days, a formal certificate from a physician becomes necessary. For most employees, the employer provides SA reimbursement for the initial 14 days. However, for other groups (e.g., unemployed individuals), the Social Insurance Agency provides reimbursements from the start of the SA-spell. Hence, data on shorter SA spells (i.e., less than 14 days) is available for these individuals in the agency’s register. In order not to introduce bias, we chose to only consider data for SA spells lasting longer than 14 days in our study.

Sickness absence can be granted for both full-time or part-time (100%, 75%, 50%, or 25%) of usual working hours. Disability pension (DP) can be provided to individuals aged between 19 and 64, if their injury or disease has resulted in a long-term or permanent reduction in work capacity. DP, like SA, can be granted either full-time or in part-time increments. Consequently, an individual on part-time DP can also receive part-time SA simultaneously. For the purposes of this study, the calculation of mean and median net days of SA were summed to whole days. For example, SA for 50% of the time over two days would be counted as one net day.

### Covariates

We categorized sociodemographic variables as follows: sex (women, men), age (age groups 18–24, 25–34, 35–44, 45–54, 55–63 years), education level (elementary school including missing data, high school 9–12 years, university or college > 12 years), country of birth (Sweden, not Sweden), degree of urbanization (cities, towns and suburbs, rural area), marital status (married, not married), and finally presence of income from work (yes, no).

For each of these variables, we chose a reference group for comparisons with new SA proportions. This selection was based on having the least expected proportion of new SA or simply being the largest group in some cases. Specifically, we chose women, individuals with university or college education level, those born in Sweden, residents in cities, unmarried individuals and those who received income from work as reference categories. Although we expected the proportion of new SA to be lower in the youngest age group (18–24), we selected the age group of 25–34 years as the reference, partly because many individuals in the younger age group would still be students and might not even be eligible for SA benefits.

### Statistical analysis

The individual’s background and sociodemographics (sex, age, level of education, country of birth, type of living area, marital status, income from work, prior TBI) and injury characteristics (type of injury, cause of injury, inpatient healthcare) were summarised using descriptive statistics and shown by SA and DP status at the time of injury.

Crude risk ratios (RR) with 95% confidence intervals were estimated via 2 × 2 cross–tabulations in SPSS, whereas adjusted RRs were obtained from modified Poisson regression with a log link and robust variance estimation (for both crude and multivariable-adjusted models). Multinomial logistic regressions were used to estimate odds ratios (OR) for associations between sociodemographic, background, and injury-related factors and the length of the SA spell. Specifically, spells were categorised as short (< 30 days), medium (30–90 days), or long (> 90 days).

For all models we excluded individuals who at the time of injury already had an ongoing SA or full-time DP, as they were not at risk of a new SA. Results from the modified Poisson models are presented as RRs (95% CIs), and results from the multinomial models as ORs (95% CIs). Statistical analyses were performed using SPSS (version 28) and SAS (version 9.4). Statistical significance was set to *p* < 0.05 and all tests were two-tailed.

## Results

We identified 2826 individuals of working age who received in- or specialized outpatient health care due to a SR-TBI within the three-year inclusion period. Table 1 shows the injury-related characteristics as well as the sociodemographic covariates. Most of the identified individuals were men, comprising 63% of the sample. The average age was 31, with the age distribution heavily skewed towards the younger age groups. A majority had either high school or university college education (81%), were born in Sweden (88%), lived in either cities or towns and suburbs (84%) and were not married (80%). The majority received income from work (75%).

**Table 1:** Sociodemographic and injury characteristics for 2826 individuals, aged 18–63 with sport-related traumatic brain injury, by sickness absence (SA) and disability pension (DP) status at the time of injury

	No New SA		New SA		Ongoing SA/DP		Total	
	*N*	%	*N*	%	*N*	%	*N*	%
Total	2375	84.0	261	9.2	190	6.7	2826	100,0
Sex								
Women	838	79.7	115	10.9	98	9.3	1051	37.2
Men	1537	86.6	146	8.2	92	5.2	1775	62,8
Age group, years								
18–24	1195	92,9	55	4.3	36	2.8	1286	45.5
25–34	584	84,9	56	8.1	48	7.0	688	24.3
35–44	290	75,9	62	16.2	30	7.9	382	13.5
45–54	205	70.4	55	18.9	31	10.7	291	10.3
55–63	101	56.4	33	18.4	45	25.1	179	6.3
Education level								
Elementary school	458	85.8	25	4.7	51	9.6	534	18.9
High school	1235	85.3	128	8.8	84	5.8	1447	51.2
University/College	682	80.7	108	12.8	55	6.5	845	29.9
Born in Sweden								
No	283	84.0	29	8.6	25	7.4	337	11.9
Yes	2092	84.0	232	9.3	165	6.6	2489	88,1
Marital status								
Not married	1963	87.1	159	7.1	132	5.9	2254	79.8
Married	412	72.0	102	17.8	58	10.1	572	20.2
Type of living area								
Big cities	922	83.7	97	8.8	82	7.4	1101	39.0
Medium-sized cities	1034	84.5	115	9.4	74	6.1	1223	43.3
Small cities/villages	419	83.5	49	9.8	34	6.8	502	17.8
Income from work								
No	593	85.1	14	2.0	90	12.9	697	24.7
Yes	1782	83.7	247	11.6	100	4.7	2129	75.3
Previous traumatic brain injury								
No	2020	84.2	236	9.8	142	5.9	2398	84.9
Yes	355	82.9	25	5.8	48	11.2	428	15.1
Cause of injury								
Transport accidents	490	72.3	121	17.8	67	9.9	678	24.0
Falls	839	83.0	78	7.7	94	9.3	1011	35.8
Strikes by objects	273	85.6	27	8.5	19	6.0	319	11.3
Strikes by living forces	773	94.5	35	4.3	10	1.2	818	28.9
Type of injury								
Concussion	2257	87.6	181	7.0	139	5.4	2577	91.2
Other intracranial injury	118	47.4	80	32.1	51	20.5	249	8.8
Inpatient healthcare								
No	1922	87.5	132	6.0	143	6.5	2197	77.7
Yes	453	72.0	129	20.5	47	7.5	629	22.3

Following the injury, a vast majority (84%) did not initiate a new SA spell, although 9% did. The remaining individuals were either already on SA (4%) or full-time DP (3%) at the time of injury. Initiating a new SA spell was relatively more common among women, older individuals, those with a higher level of education, those who were married, and those who had income from work. Finally, it was found that 15% of the injured individuals had previous healthcare visits (greater than 1 year ago) for TBI. Initiating a new SA spell was relatively more common among individuals without a previous TBI (10%) as compared to those with a previous TBI (6%).

The main diagnosis for the vast majority (91%) of individuals was concussion (S06.0). Falls were the most common cause of injury (36%), followed by strikes by living forces (29%), and transport accidents (24%). The least common cause of injury was strikes by objects (11%). Most of the individuals (78%) did not require inpatient healthcare and was treated only in specialized outpatient healthcare settings. Initiation of a new SA spell was more common among those with other forms of TBI (32%) compared to individuals with concussion (7%). A new SA Spell was also more common among those individuals who were injured in transport accidents (18%), whereas those injured by strikes of living forces represented the least common injury mechanism that were followed by a new SA (4%). Initiation of a new SA spell was more frequent among those who had received inpatient healthcare (21%) compared to those who received specialized outpatient care only (6%).

Once we excluded individuals who were either on ongoing SA or full-time DP at the time of the injury, a total of 2636 individuals remained. These remaining individuals, who were at risk for a new SA, were included in the analysis of risk ratios (see Table 2). The crude risk ratios indicated an increased risk for new SA among women, older age groups, individuals with higher educational level, those who were not married, and those who had income from work. Also, individuals without a previous TBI had a higher risk as compared to those with a previous TBI. In the fully adjusted model, these sociodemographic and background factors did not reach significance, except for lower risk (adjusted RR 0.27, 95% CI 0.16–0.47) for those who did not have income from work.

**Table 2:** Crude risk ratios (RR) and mutually adjusted risk ratios (RR) with 95% confidence intervals (CI) for new sickness absence (SA) following a sport-related traumatic brain injury, by sociodemographic and injury characteristics among the 2636 injured individuals without already ongoing SA or full-time disability pension

	*N*	% new SA	Crude RR (95% CI)	Adjusted RR (95% CI)
Sex				
Women	953	12.1	1.39 (1.10–1.75)	1.10 (0.89–1.37)
Men	1683	8.7	ref.	ref.
Age group, years				
18–24	1250	4.4	0.50 (0.35–0.72)	0.67 (0.47–0.96)
25–34	640	8.8	ref.	ref.
35–44	352	17.6	2.01 (1.44–2.82)	1.31 (0.93–1.84)
45–54	260	21.2	2.42 (1.72–3.41)	1.29 (0.90–1.84)
55–63	134	24.6	2.81 (1.91–4.15)	1.27 (0.84–1.94)
Education level				
Elementary school	483	5.2	0.40 (0.25–0.58)	0.97 (0.64–1.48)
High school	1363	9.4	0.69 (0.54–0.87)	1.05 (0.83–1.32)
University/College	790	13.7	ref.	ref.
Born in Sweden				
No	312	9.3	0.93 (0.65–1.35)	0.95 (0.68–1.32)
Yes	2324	10.0	ref.	ref.
Marital status				
Not married	2122	7.5	0.38 (0.30–0.48)	0.79 (0.61–1.03)
Married	514	19.8	ref.	ref.
Type of living area				
Big cities	1019	9.5	ref.	ref.
Medium-sized cities	1149	10.0	1.05 (0.81–1.36)	0.97 (0.76–1.24)
Small cities/villages	468	10.5	1.10 (0.79–1.52)	1.03 (0.75–1.40)
Income from work				
No	607	2.3	0.19 (0.11–0.32)	0.27 (0.16–0.47)
Yes	2029	12.2	ref.	ref.
Previous traumatic brain injury				
No	2256	10.5	ref.	ref.
Yes	380	6.6	0.63 (0.42–0.94)	0.95 (0.64–1.39)
Cause of injury				
Transport accidents	611	19.8	4.57 (3.19–6.56)	2.14 (1.43–3.19)
Falls	917	8.5	1.96 (1.33–2.89)	1.13 (0.75–1.69)
Strikes by objects	300	9.0	2.08 (1.28–3.37)	1.66 (1.03–2.69)
Strikes by living forces	808	4.3	ref.	ref.
Type of injury				
Concussion	2438	7.4	ref.	ref.
Other intracranial injury	198	40.4	5.44 (4.37–6.78)	2.76 (2.15–3.53)
Inpatient healthcare				
No	2054	6.4	ref.	ref.
Yes	582	22.2	3.45 (2.76–4.32)	1.92 (1.50–2.46)

However, all the factors related to the injury itself were associated with higher odds of a new SA in the fully adjusted model. Specifically, those who had received inpatient healthcare had an adjusted RR of 1.92 (95% CI 1.50–2.46) for new SA compared with those treated only as outpatients. Individuals with other intracranial injuries had an adjusted RR of 2.76 (95% CI 2.15–3.53) compared to those with concussion. The mechanism of injury also played a significant role: compared with injuries sustained by strikes from living forces, transport accidents carried an adjusted RR of 2.14 (95% CI 1.43–3.19) and strikes by objects an adjusted RR of 1.66 (95% CI 1.03–2.69) for new SA.

Among all individuals at risk, approximately 10% incurred a new SA. The duration of these new SA episodes varied, with 3.1% encountering a short-term SA of fewer than 30 days, 3.5% experiencing a medium-term SA spanning 30 to 90 days, and 3.3% having a prolonged SA exceeding 90 days. Notably, individuals with other forms of TBI than concussions and those who received inpatient healthcare were significantly more likely to have medium-term and long-term SA. Moreover, transport accidents were associated with an increased likelihood of medium-term SA, a trend also observed among those struck by objects. Age also influenced SA duration, with older groups trending toward longer spells, though this was not uniformly statistically significant. Detailed odds ratios and confidence intervals are presented in Table 3.

**Table 3:** Multinomial logistic regression showing crude and mutually adjusted odds ratios (OR) for all factors and confidence intervals (CI) for different duration of new sickness absence (SA) following a sport-related traumatic brain injury, by sociodemographic and injury characteristics among the 2636 injured individuals without already ongoing SA or full-time disability pension

	SA < 30 Net Days		SA 30–90 Net Days		SA > 90 Net Days	
	Crude	Adjusted	Crude	Adjusted	Crude	Adjusted
Sex						
Women	1.44 (0.92–2.24)	1.23 (0.77–1.98)	1.33 (0.87–2.02)	0.94 (0.60–1.49)	1.60 (1.04–2.46)	1.32 (0.81–2.17)
Men	ref.	ref.	ref.	ref.	ref.	ref.
Age group, years						
18–24	0.45 (0.25–0.81)	0.62 (0.33–1.17)	0.51 (0.27–0.97)	0.68 (0.35–1.34)	0.49 (0.23–1.03)	0.70 (0.31–1.56)
25–34	ref.	ref.	ref.	ref.	ref.	ref.
35–44	1.84 (1.00–3.38)	1.23 (0.63–2.39)	2.12 (1.11–4.03)	1.28 (0.64–2.58)	3.02 (1.51–6.03)	1.74 (0.79–3.81)
45–54	1.73 (0.88–3.43)	1.10 (0.52–2.33)	2.40 (1.21–4.75)	1.23 (0.58–2.61)	5.09 (2.59–9.98)	2.27 (1.03–5.02)
55–63	0.75 (0.22–2.56)	0.47 (0.13–1.70)	5.48 (2.78–10.80)	2.43 (1.08–5.46)	4.96 (2.23–11.02)	1.39 (0.51–3.76)
Education level						
Elementary school	0.36 (0.17–0.79)	0.92 (0.39–2.16)	0.48 (0.25–0.94)	1.26 (0.58–2.73)	0.20 (0.08–0.50)	0.67 (0.24–1.92)
High school	0.69 (0.43–1.10)	0.99 (0.59–1.66)	0.66 (0.42–1.03)	1.04 (0.62–1.73)	0.63 (0.40–0.98)	1.19 (0.70–2.03)
University/College	ref.	ref.	ref.	ref.	ref.	ref.
Born in Sweden						
No	1.03 (0.52–2.01)	1.00 (0.49–2.03)	0.79 (0.39–1.59)	0.85 (0.40–1.81)	0.97 (0.50–1.90)	0.98 (0.46–2.11)
Yes	ref.	ref.	ref.	ref.	ref.	ref.
Marital status						
Not married	0.35 (0.22–0.55)	0.58 (0.33–1.02)	0.35 (0.23–0.54)	0.83 (0.48–1.43)	0.29 (0.19–0.45)	0.75 (0.42–1.35)
Married	ref.	ref.	ref.	ref.	ref.	ref.
Type of living area						
Big cities	ref.	ref.	ref.	ref.	ref.	ref.
Medium-sized cities	1.13 (0.69–1.84)	1.13 (0.68–1.87)	1.26 (0.78–2.05)	1.11 (0.66–1.86)	0.85 (0.53–1.34)	0.74 (0.44–1.26)
Small cities/villages	1.03 (0.54–1.96)	1.14 (0.59–2.24)	1.75 (1.00–3.05)	1.47 (0.80–2.71)	0.70 (0.36–1.34)	0.59 (0.28–1.24)
Income from work						
No	0.20 (0.08–0.48)	0.27 (0.10–0.71)	0.17 (0.07–0.42)	0.19 (0.07–0.50)	0.15 (0.05–0.40)	0.17 (0.06–0.51)
Yes	ref.	ref.	ref.	ref.	ref.	ref.
Previous traumatic brain injury						
No	ref.	ref.	ref.	ref.	ref.	ref.
Yes	0.62 (0.29–1.29)	0.80 (0.37–1.69)	0.61 (0.30–1.22)	0.95 (0.46–1.97)	0.58 (0.28–1.22)	1.12 (0.50–2.49)
Cause of injury						
Transport accidents	2.86 (1.54–5.32)	1.72 (0.86–3.44)	11.72 (5.28–26.01)	5.96 (2.54–14.01)	5.26 (2.73–10.12)	1.60 (0.73–3.49)
Falls	1.38 (0.73–2.62)	0.97 (0.49–1.92)	3.29 (1.42–7.65)	1.72 (0.71–4.21)	2.23 (1.13–4.39)	0.72 (0.33–1.60)
Strikes by objects	2.30 (1.09–4.85)	1.82 (0.84–3.90)	3.64 (1.34–9.87)	2.96 (1.07–8.21)	1.18 (0.41–3.38)	0.85 (0.28–2.64)
Strikes by living forces	ref.	ref.	ref.	ref.	ref.	ref.
Intracranial injury						
Concussion	ref.	ref.	ref.	ref.	ref.	ref.
Other intracranial injury	1.79 (0.81–3.96)	1.46 (0.61–3.45)	8.24 (5.10–13.32)	4.06 (2.26–7.28)	20.99 (13.23–33.32)	10.20 (5.80–17.95)
Inpatient healthcare						
No	ref.	ref.	ref.	ref.	ref.	ref.
Yes	1.76 (1.08–2.86)	1.59 (0.93–2.72)	4.34 (2.85–6.59)	2.19 (1.34–3.60)	8.79 (5.53–13.96)	3.68 (2.10–6.46)

## Discussion

To our knowledge, this is the first nationwide register study of SA following SR-TBIs in the general adult population. Of all the SR-TBIs we studied, 91% were concussions, and 78% received only specialized outpatient healthcare treatment. Our study primarily analyzed the incidence of new SA spells after injury in individuals at risk (i.e., individuals not already on SA or full-time DP). We discovered significant differences in the rate of new SA based on factors related to the injury. First, concussions resulted in a new SA in only 7% of the cases. In contrast, other intracranial injuries led to a new SA in 40% of the cases. Second, among those who did not require inpatient healthcare only 6% had a new SA, while 22% of those who received inpatient healthcare had a new SA following their injury. Third, and finally, among the SR-TBIs we analysed, those resulting from transport accidents such as bicycling and horseback riding led to a new SA in 20% of the cases. In comparison, injuries from falls or from being struck by objects was associated with a 9% new SA rate, while those stemming from strikes by living forces resulted in a new SA in just 4% of the cases. These findings highlight the varied rates of new SA depending on the specifics of the SR-TBI and treatment received.

Our study found low levels of prolonged SA spells: only 3.3% of the entire SR-TBI cohort had an SA spell exceeding 90 days, with individuals suffering from concussions at an even lower rate of 1.7%. These figures contrast sharply with the broader research trends, such as those highlighted in our introduction. For instance, previous systematic reviews [25, 26], have reported up to 20% of mild TBI patients not successfully returning to work six months after injury. Several factors might explain this discrepancy. Our cohort’s relatively healthy, active lifestyle could contribute to lower SA rates, as these individuals likely possess greater health resilience compared to the general population included in other studies. Furthermore, our reliance on high-quality register data minimizes the selection biases often present in clinical studies, potentially leading to more accurate representations of SA durations. However, it is important to acknowledge that our register-based approach is constrained by the available data, and strict eligibility for sickness absence compensation after an injury can only be inferred, not directly observed, as discussed in more detail below.

Our analysis of sociodemographic variables traditionally associated with SA in the medical literature revealed a distinct pattern for SR-TBI cases. Except for age, where higher age was linked to higher odds of prolonged SA, factors such as sex, education and type of living area did not significantly predict the likelihood of SA after injury. Our findings hint that, for this specific population, the nature of the injury itself might be the primary determinant of recovery and return to work. It is worth noting that engagement in sporting activities requires a baseline level of health and fitness, such as the physical ability to ride a bike or participate in team sports. This requisite level of pre-injury health could indicate that the SR-TBI group are intrinsically healthier and more resilient, making them less vulnerable to the typical influences tied to sociodemographic variables. This is supported by Kjeldgård et al. [32] who found lower excess SA among bicyclists compared to pedestrians and car occupants after road traffic accidents, which may reflect a generally better health of bicyclists.

In our study, we observed that those injured in sport-related transport accidents had a higher likelihood of a new SA when compared with individuals injured in other kinds of sports. The pronounced mechanical forces inherent in these accidents may account for this pattern, as high-energy impacts are more likely to result in multifaceted injuries, including fractures. Fracture injuries typically heal within a few months and would thus likely only increase short to medium-term SA, aligning with our finding where the cause of injury no longer held a significant effect for SA spells exceeding 90 days.

In our findings, the presence of income for work was consistently associated with higher risk of a new SA. This correlation is primarily reflecting the eligibility criteria within the Swedish social insurance system, where individuals must have had an income from work for at least six months to qualify for SA benefits. Considering that our study population skewed towards younger age groups many may not have yet accumulated the requisite work history to become eligible for SA, which would skew SA rates downwards for this group. Therefore, lower incidence of SA among those without registered income from work could mask the true incidence of individuals failing to return to work after injury in less economically established populations.

Our study shows the use of SA after SR-TBI, which should not be confused with the actual need of SA. Wäljas et al. [33] found that, despite similar neuropsychological status and self-reported post-concussive symptoms, mild TBI patients exhibiting intracranial signs of injury returned to work slower compared to those without such signs (i.e., concussion). Wäljas et al. suggests that different sick-listing practises may be at play, with physicians potentially more inclined to issue certificates to patients with observable signs of injury. Furthermore, a significant number of mild TBI patients who have resumed work continue to experience persistent symptoms, as reported in various studies [34]. Researchers interpret this phenomenon as behavioural adaptation rather than a genuine return to pre-injury health [35]. However, this adaptation could come at a cost. Silverberg et al. [36] discovered that mild TBI patients who had returned to work by three months still endorsed numerous post-concussive symptoms and acknowledged reduced efficiency and increased mistakes. Future studies should evaluate work capacity as well as return to work status to capture more subtle effects of SR-TBI.

### Strengths and limitations

The strength of this study is the use of high-quality national registers. All residents in Sweden meeting the inclusion criteria were eligible for the study, rather than just a selected sample. This comprehensive inclusion enabled detailed analysis of various subgroups. We avoided the potential biases associated with self-reported data. Instead, all data were systematically registered by authority administrators or healthcare staff. There were no dropouts.

Our study presents several limitations that merit discussion. Firstly, our dataset contains only SA spells exceeding 14 days, omitting shorter durations. In addition, we excluded individuals with a TBI diagnosis in the preceding 365 days to minimize confounding from earlier injuries. As such, our results primarily reflect outcomes following single-event TBIs. While this design choice improves internal validity, it may limit generalizability to individuals with repeated TBIs — a subgroup that may be at greater risk of prolonged SA. Secondly, the Swedish national patient registers account for inpatient and specialized outpatient healthcare, but exclude primary healthcare services, such as those provided by general practitioners. While no national data are available on how many sport-related TBIs are managed solely in primary care in Sweden, international studies suggest that this may represent a substantial proportion. For example, the BIONIC study from New Zealand found that 36% of TBI cases were not captured in hospital records — 8% were seen only in primary care, and 28% were identified through other non-hospital sources [37]. In addition, Sosin et al. [38] estimated that 14% of individuals with TBI in the United States were treated in outpatient settings such as clinics or physicians’ offices — that is, in primary care rather than specialized outpatient or emergency care — and that 25% did not seek medical care at all. As these milder cases are likely to involve shorter or no sickness absence, our study may underestimate the total burden of sickness absence following sport-related TBI, particularly short-term spells. These constraints likely result in an underrepresentation of milder cases, but also focus the study on the more clinically significant injuries — those most likely to result in extended sickness absence. Moreover, our classification of sickness absence duration was based on net days (i.e., the summation of part-time absence to full-day equivalents). A comparison with gross days, which include all calendar days with any level of SA, showed that 12 additional individuals would have been classified as having long-term SA (> 90 days), increasing the proportion from 3.3 to 3.7%. This suggests that part-time SA had limited impact on our duration-based classification. Thirdly, the reliability of the registers’ hinges on the quality of data input, and some level of misclassifications of injury-related data is expected. Finally, the registers don’t capture specific details like the exact sport involved or its competitive level (e.g., recreational, elite, professional).

## Conclusion

The majority of the SR-TBI cases were concussions, which were less likely to lead to SA, compared to other forms of TBI. Our analysis revealed that both the nature of the injury and the treatment received were key determinants of the likelihood of new SA, with transport accidents particularly associated with higher SA rates. Notably, while the specifics of the injury played a significant role in the outcomes observed, sociodemographic factors did not. To conclude, most adults recovering from SR-TBI, especially those who only require outpatient healthcare, do not experience prolonged SA, highlighting the resilience of individuals with less severe injuries like concussions.

## Data Availability

Due to stringent privacy regulations, we are unable to make this data publicly available. In compliance with the General Data Protection Regulation (EU) 2016/679, Swedish law (SFS 2018:218), the Swedish Data Protection Act (2018:218), the Swedish Ethical Review Act (2003:460), and the Public Access to Information and Secrecy Act (2009:400), data can only be shared after a legal review, and solely with researchers who meet the established criteria for accessing this sensitive and confidential data. Readers may contact Professor Kristina Alexanderson (kristina.alexanderson@ki.se) regarding the data.

## Abbreviations

CI: Confidence Intervals
CT: Computed Tomography
DP: Disability Pension
ICD-10: International Statistical Classification of Diseases and Related Health Problems, tenth revision
LISA: Longitudinal Integration Database for Health Insurance and Labour Market Studies
MiDAS: Micro Data for Analysis of the Social Insurance
MR: Magnetic Resonance
OR: Odds Ratios
SA: Sickness absence
SAS: Statistical Analysis System
SPSS: Statistical Package for the Social Sciences
SR-TBI: Sport-Related Traumatic Brain Injury
TBI: Traumatic brain injury
